# Determination of Sediment Oxygen Demand in the Ziya River Watershed, China: Based on Laboratory Core Incubation and Microelectrode Measurements

**DOI:** 10.3390/ijerph13020232

**Published:** 2016-02-19

**Authors:** Nan Rong, Baoqing Shan, Chao Wang

**Affiliations:** 1 State Key Laboratory on Environmental Aquatic Chemistry, Research Center for Eco-Environmental Science, Chinese Academy of Science, Beijing 100085, China; rongnan420@163.com; 2 University of Chinese Academy of Science, Beijing 100049, China; 3 Changjiang Water Resource Protection Institution, Wuhan 430051, China; cwwhu@163.com

**Keywords:** sediment oxygen demand, microelectrode, microprofile, oxygen flux, the Ziya River Watershed

## Abstract

A study coupling sedimentcore incubation and microelectrode measurementwas performed to explore the sediment oxygen demand (SOD) at 16 stations in the Ziya River Watershed, a severely polluted and anoxic river system in the north of China. Total oxygen flux values in the range 0.19–1.41 g/(m^2^·d) with an average of 0.62 g/(m^2^·d) were obtained by core incubations, and diffusive oxygen flux values in the range 0.15–1.38 g/(m^2^·d) with an average of 0.51 g/(m^2^·d) were determined by microelectrodes. Total oxygen flux obviously correlated with diffusive oxygen flux (*R*^2^ = 0.842). The microelectrode method produced smaller results than the incubation method in 15 of 16 sites, and the diffusive oxygen flux was smaller than the total oxygen flux. Although the two sets of SOD values had significant difference accepted by the two methods via the Wilcoxon signed-rank test (*p* < 0.05), the microelectrode method was shown to produce results that were similar to those from the core incubation method. The microelectrode method, therefore, could be used as an alternative method for traditional core incubation method, or as a method to verify SOD rates measured by other methods. We consider that high potential sediment oxygen demand would occur in the Ziya River Watershed when the dissolved oxygen (DO) recovered in the overlying water.

## 1. Introduction

Dissolved oxygen (DO) is one of the most important indicators of water and ecosystem quality in rivers, channels, reservoirs, and lakes. Low DO concentrations, or in extreme cases, anaerobic conditions, in a normally well oxygenated river system represent severe threats to fish survival, blackwater, and odors [1,2]. The variability of DO in rivers is caused by the influence of either sources or sinks of oxygen. Re-aeration from the atmosphere and photosynthesis are the major sources of DO. Sediment oxygen demand (SOD) is a major oxygen sink, in addition to respiration by aquatic plants and the oxidation of organic materials and other reduced matters in the water column. SOD has been defined as the rate of oxygen consumption, biologically or chemically, on or in the sediment at the bottom of a water body [3]. SOD encompasses both the respiration rates of benthic communities and the chemical oxidation of reduced substances in the sediment [4]. SOD has been found to contribute significantly to oxygen depletion. In some water systems, SOD can account for more than half of the total oxygen demand and play a primary role in the water quality [5,6,7,8]. It is therefore essential to estimate the oxygen demand exerted by sediments.

Although the SOD value may represent a potentially major effect on the total oxygen demand in a water system, a universally accepted standard and a uniform procedure for SOD measurement have not yet been agreed [9]. Bowman and Delfino [10] have compiled an excellent review and comparison of laboratory and *in situ* methods for measuring SOD. To assess this critical sink of DO in water bodies, researchers conventionally use two approaches: first, measuring changes in oxygen concentration in the overlying water of enclosed benthic systems, including sediment core incubation in the laboratory and *in situ* benthic chamber deployment, providing a direct measurement of total oxygen flux; and second, determining the oxygen concentration profile in the sediment using microelectrodes, providing a measurement of the diffusive oxygen flux [11,12,13]. Estimates of SOD produced by the two techniques have been reported but show contrasting results. The total oxygen uptake was found to be in excess of the diffusive oxygen flux by a factor of 2–3 on the Washington shelf [14]. A two-fold difference between the total oxygen flux and the diffusive oxygen flux was found in the saline Lake Grevelingen [15]. Conversely, a reasonable agreement between the two techniques was found on the Pacific slope and rise [16]. By comparing data and results from one method with another method would be a useful validating technique, although this is not common.

Over the past few decades, more than half of the major rivers in China have transformed from clear and potable to malodorous blackwater with fish extinctions. The Ziya River Watershed, located in the Jing-Jin-Ji area, that includes Beijing, Tianjin, and Hebei in northern China, is one of the most malodorous blackwater watersheds and is now in a state of severe hypoxia.The mean concentration of DO in rivers is below 3 mg/L–less than 2 mg/L in many reaches close to cities—indicating that DO was apparently a limiting factor of river ecosystem health. Over the past decades, considerable attention (e.g., the Water Pollution Control and Treatment National Science and Technology Major Project) has been directed toward the oxygen consumption effects of chemical oxygen demand and ammonia in overlying waters in streams of the Ziya River Watershed. Until recently, little study has directly addressed the hypoxia caused by the sediment oxygen consumption. Research on the sediment oxygen demand will be of great help for water pollution control and sediment restoration in the Ziya River Watershed.

Although the laboratory core incubation and DO microprofile have been used regularly to detect the SOD in rivers, there has been little exploration of the sediment oxygen consumption rates in the Ziya River Watershed. In this study, using simultaneous flux estimates from the two techniques of laboratory core incubations and microelectrodes, we determined the sediment oxygen demand. We compare SOD based on the oxygen flux data and other data from the literature. The primary purpose of this study is to determine the potential degree and the spatial variation of SOD in the Ziya River Watershed, using the laboratory core incubation and microprofile methods. The DO consumption curves and DO microprofiles would be obtained. Processes related to these two determinations will also be discussed in this paper. The secondary objective of this study is to test the accuracy of the measured SOD rates by validating core incubation measurements with microprofile measurements.The results of SOD would be directive and practical important, especially for heavy water pollution control.

## 2. Materials and Methods

### 2.1. Study Area

**Figure 1:**
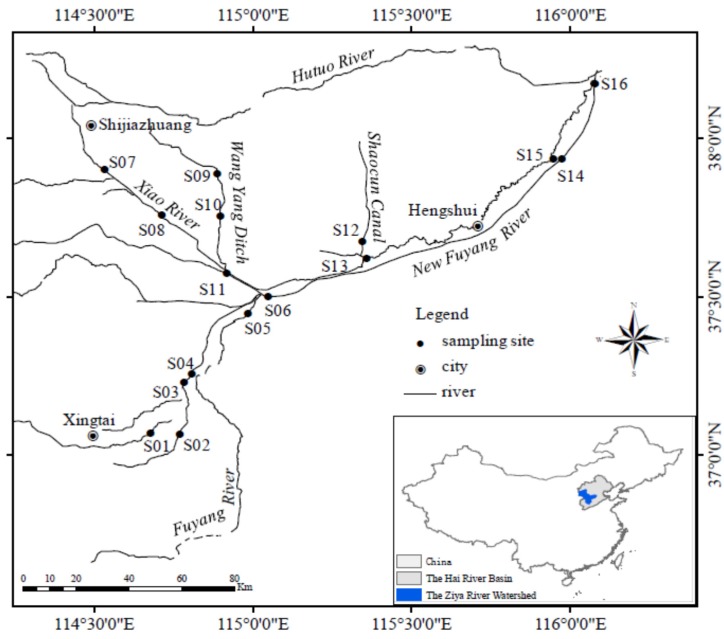
Location of the study sites in the Ziya River Watershed, China.

The Ziya River Watershed is located in the south of the Hai River Basin, China (Figure 1). The regional water resources in the Hai River Basin are deficient innately. Coupled with the rapid development of the regional economy and water pollution, especially influencing the oxygen consumption, pollution in the Hai River Basin is more serious than in the “three rivers” (Yangtze River, Yellow River, and Pearl River) in China. In terms of water resources and water environmental problems, the Ziya River Watershed is one of the most seriously affected watersheds in the Hai River Basin. The Ziya River Watershed is the epitome of ecological environment in the Hai River Basin. It is bounded on the west by the Taihang Mount, on the east by the Bohai Sea, on the north by the Daqing River Watershed, and on the south by the Zhangwei River Watershed. It stretches across Shanxi Province, Hebei Province, and Tianjin, and its 27.4 thousand square kilometers drainage area supports a population of 19 million. The two main stems of the Ziya River Watershed are the Hutuo River and the Fuyang River.

Severe hypoxia, blackwater, and odors are the dominant characteristics of the Ziya River Watershed. The plain area of the Ziya River Watershed exhibits particularly severe DO depletion. Most of the rivers in the watershed are turbid and contain blackwater. According to the Environmental Quality Standards for Surface Water in China, the DO concentration is worse than Class IV water standard (the content is lower than 2.0 mg/L). Water quality standards typically require O_2_ > 4.8 mg/L to protect aquatic life. High levels of ammonia, chemical oxygen demand, and organics in the water column, originating from the discharge of municipal sewage treatment plants, were already regarded as important factors of severe DO depletion. The Ziya River Watershed also has highly polluted sediments and sludge deposits.

We established 16 sampling sites (Figure 1) in the plain area of the Ziya River Watershed to measure SOD in August 2013. Sites S01–S05 were located at Niuwei River and Sha River around Xingtai in the upstream part of the Ziya River Watershed. Xingtai has well-developed agricultural and papermaking industries. Agricultural non-point pollution and papermaking wastewater often represent a problem for the natural river system. Sites S06–S11 were located at Xiao River and Wang Yang Ditch around Shijiazhuang in the midstream part of the Ziya River Watershed. Shijiazhuang is the provincial capital of Hebei, and has the highest Gross Domestic Product in the province. The pharmaceutical industry is the dominant industry of the Shijiazhuang economy. Pharmaceutical effluents usually cause high chemical oxygen demand which has a harmful effect on the rivers. Sites S12–S13 were located at Shaocun Canal, and sites S14–S16 were located at the Fuyang and New Fuyang Rivers. These sites are located around Hengshui and in the downstream part of the Ziya River Watershed. The Shaocun Canal generally admits leather manufacturing wastewater from Shijiazhuang, and the concentration of heavy metals in the water and sediments is relatively high. The Fuyang River and New Fuyang River mainly receive agricultural non-point source pollutants, and there are no large industries nearby.

### 2.2. Sampling

Sediment samples from the top 20 cm of the riverbed were taken using a dredge sampler at each site. The top 20 cm represents the most biologically active depositional layer in relatively slow flowing streams [17]. Three parallel samples were collected at each site. Sediment samples were stored in polyethylene tubes, with air-isolating cocks, to reduce the likelihood of sediment oxidization during the preservation period. Sediment samples were placed in refrigerators at 4 °C (a representative preservation temperature). The samples were brought to the laboratory immediately after retrieval and incubations generally startedwithin 4 h after sampling.

### 2.3. Laboratory Core Incubation Measurements

As a result of the resistance of mass transfer, the underlying sediment contributes little to SOD, and SOD is only controlled by the surface 0.5 cm thick sediment [18]. In general, SOD could be accurately measured by core incubation when the sediment thickness is more than 10 cm. In this study, sixteen 6-cm-diameter Perspex cores (28 cm height) were used for incubations. Before incubation, a Perspex tube was inserted to a polyethylene tube with 10cm depth. Then the sediment depth in the incubation tube was 10 cm. The overlying water was replaced by tap water before the incubation (14 cm thickness of overlying water). Three blank experiments showed that there were few oxygen consumption substances in the tap water. Sediment cores were kept at *in situ* temperature of 25 °C. The DO concentration in the overlying water of each core was recorded manually every two hours using 16 portable hand-held DO meters. These DO meters were attached to rubber plugs and placed in the core center, ensuring a well-mixed DO concentration in the overlying water. The change in oxygen concentration with time in the cores was linear after an initial period, usually less than 15 min, in which the change in oxygen concentration can be nonlinear and more rapid. This nonlinearity is usually attributed to the suspension of some sediment during replacement of the overlying water; this sediment then settles back to the bottom within the first few minutes after the rubber plugs are fixed. For this reason, DO concentration recordings were conducted 15 min after closure of the rubber plugs. Subsequently, sediment cores were incubated in the dark for 24 h until DO concentration in the overlying water dropped to 2.0 mg/L. The rates of oxygen consumption were calculated from the slopes along DO *versus* time profiles and the area of the sediment–water interface and the SOD were expressed in g/(m^2^·d). Mathematically, the core incubation SODrate can be formulated as:

(1) $${\text{SOD}}_{T}=\left(24S\times V\right)/10^{3}A$$

where SOD*_T_* is the sediment oxygen demand at T °C, g/(m^2^·d); *S* is the slope of the linear portion of the usage curve, mg/(L·h); *V* is the volume of the overlying water, L; and *A* is the area of the bottom sediment, m^2^. Subtracting the two rubber plug thickness of 4 cm, the *V*/*A* ratio in the Perspex core was 140 L/m^2^. Thus, the specific formula for the core incubator in this study is:

(2) $${\text{SOD}}_{T}=3.360S$$

SOD at temperature T is usually adjusted to a SOD rate at 20 °C. Existing studies have proven that temperature has an effect on sediment oxygen demand. Some limited experimental laboratory research has explored a temperature correction factor for sediment oxygen demand; Baity [19] applied a modified Arrhenius equation for SODrates. The form of the equation is:

(3) $${\text{SOD}}_{T}={\text{SOD}}_{T\text{ref}}\theta^{\left(T-20\right)}$$

where the most frequently used *T*ref is 20 °C and θ is defined as a constant for which a value of 1.065 is commonly used [20]. This equation provides the temperature dependence of a reaction based on its activation energy.

### 2.4. Microelectrode Measurements

In this study, we constructed a sediment oxygen measuring system which was composed by a gold amalgam microelectrode (manufactured by Wang *et al.* [21]), a micromanipulator (Narishige Co., Ltd.: Tokyo, Japan), and a CHI 660 electrochemical analyzer (CH Instruments Inc.,: Texas, TX, USA). The microelectrode was of the standard three-electrode system with the Hg-Au microelectrode as the working electrode, the Pt wire as the counter-electrode, and the solid Ag/AgCl electrode as the reference electrode. The oxygen microelectrode was with a tip diameter of 100 μm, response time <2 s and stirring sensitivity <2%. The micromanipulator controlled the electrode tip to move downward at a minimal step of 0.01 mm. The vertical distribution of DO can be measured with a minimum resolution of 0.03 mm.There were no considerable differences in magnitude between the oxygen flux obtained by this device and by other studies, showing the reliability of the method [22,23]. Further details of the experimental protocol were described in Wang *et al.* [21].

A mixture of 70 g of sediment with 200 mL deionized water was combined in a 400-mL beaker, first removing large plant residues and debris to prevent microelectrodes from breaking off during the oxygen profile measurement. The mixture of sediment and water was stirred until completely uniform, then settled quickly and incubated for two days at 25 °C. After the incubation, a new and distinct sediment–water interface was formed that was suitable for the oxygen profile measurement. During these experiments, microelectrode measurements of 16 vertical oxygen distributions were acquired using the microprofiler. Based on the zero-order kinetics oxygen profile model, formulas calculating diffusive oxygen flux across the sediment-water interface were derived. The diffusive oxygen flux (*J*) across the sediment–water interface can be calculated using Fick’s first law of diffusion [22,24]:

(4) J=φ·Ds·(dC/dz),(z=0)

where φ is the porosity of the sediment, *Ds* is the pore water diffusion coefficient and *dC/dz* is the vertical oxygen concentration gradient at the sediment–water interface. Such calculations of diffusive fluxes based on measured oxygen profiles of gases and ions are common in aquatic science [25,26,27].

The DO microprofile was simulated under steady-state conditions. Under steady-state conditions and negligible advection, the oxygen profile in the pore water of sediment results from a balance between diffusion and reaction such that:

(5) $$\frac{d}{dz}\left(\varphi \cdot Ds\cdot \frac{dc}{dz}\right)-R_{O_{2}}=0$$

where $R_{O_{2}}$ is the oxygen consumption rate, and is most often expressed by first- or zero-order kinetics. We solved the equation for zero-order kinetics (the zero-order reaction model was more suitable for the sediments, see Wang *et al.* [21]).

For zero-order kinetics, Equation (5) becomes:

(6) $$\varphi \cdot Ds\cdot \frac{d^{2}c}{dz^{2}}=k_{0}$$

Considering the boundary conditions,:

(7) $$c=c_{0}e^{-z\sqrt{k}}$$

(8) $$c=\frac{1}{2}kz^{2}-\sqrt{2c_{0}k}\times z+c_{0}$$

The measured DO microprofile was simulated using Equations (7) and (8) in Origin 8.5 using nonlinear fitting tools. Then dc/dz can be obtained by first derivation of the oxygen profile equation.

The sediment porosity of each sample was measured when the oxygen profiling was complete. It was calculated using the following formula [28]:

(9) $$\varphi =V_{PW}/V_{b}$$

where *V_PW_* was the volume of pore water and *V_b_* was the bulk volume of the sediment.

The diffusion coefficient in sediment pore water (*Ds*) can be derived from [24]:

(10) $$Ds=Dm\left(1-\ln\varphi^{2}\right)$$

where *Dm* is the molecular diffusion coefficient in water.

## 3. Results and Discussion

### 3.1. SOD Determined by Core Incubations

**Figure 2:**
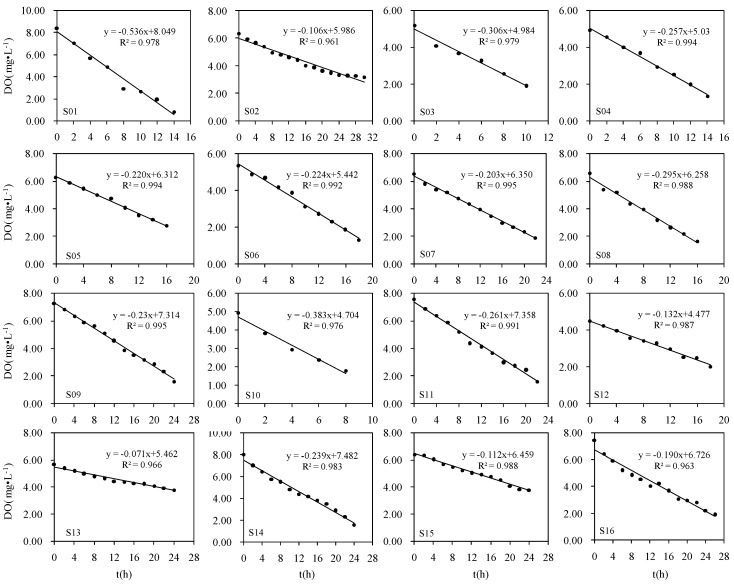
The DO depletion profiles in water overlying the sediment measured by laboratory core incubations during August, 2003 at 16 sampling stations in the Ziya River Watershed.

The DO depletion profiles in water overlying the sediment samples collected from the 16 sampling stations are graphically presented in Figure 2. Time zero represents the beginning of the experimental period. DO in the overlying water declined to 2 mg/L in 24 h for most cores, with the exception of some cores that took more than 30 h to decline below this level. The correlation coefficients (*R*^2^) were in the range 0.961–0.995. The rates of oxygen consumption were calculated from the slopes along the DO *versus* time profiles and the area of the sediment–water interface.

**Figure 3:**
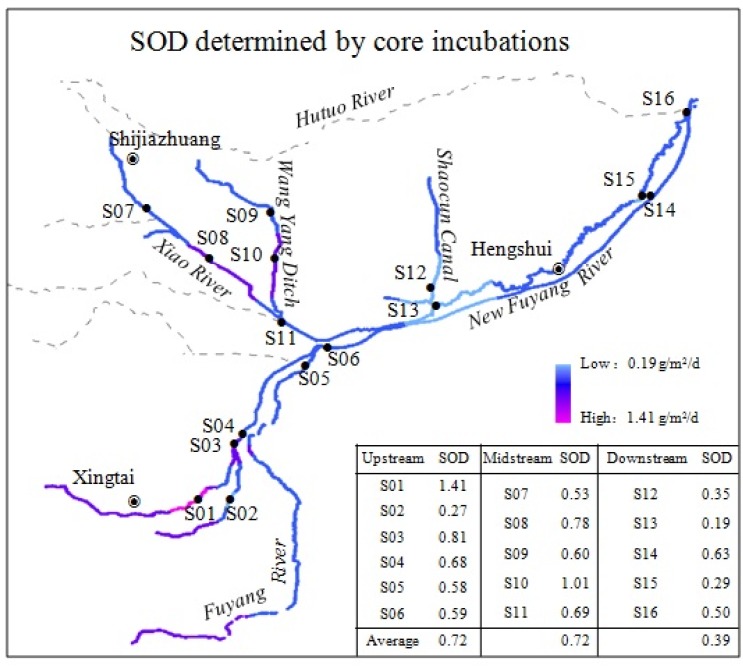
Spatial variations of the sediment oxygen demanddetermined by laboratory core incubations in the Ziya River Watershed.

Spatial variations of the SOD determined by laboratory core incubations are presented in Figure 3. SOD rates measured in the laboratory corrected to 20 °C ranged from 0.19 to 1.41 g/(m^2^·d), with an average of 0.62 g/(m^2^·d). Maximum SOD was found at S01 and minimum SOD was found at S13, with a factor of 7.5 between the extremes. Spatial variation of SOD in the Ziya River watershed was very apparent. High levels of SOD were presented in the upstream and the midstream parts of the watershed, and the SOD in the downstream was relatively low. The average flux in the upstream was 0.72 g/(m^2^·d), which was the same as the average flux in the midstream but higher than that in the downstream (0.39 g/(m^2^·d)). Many capillary tributaries in the upstream and the midstream parts of the watershed widely admitting domestic and industrial wastewater may be the causes of the higher SOD in these areas.

### 3.2. SOD Determined by Microelectrodes

**Figure 4:**
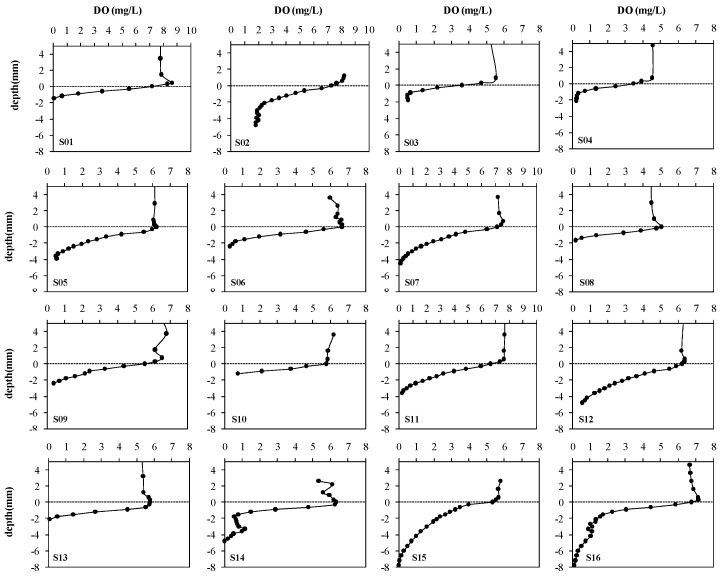
Sixteen microprofiles of dissolved oxygen in the sediment measured by microelectrodes during August 2003 at16sampling stations in the Ziya River Watershed.

Sixteen oxygen microprofiles were performed during this microelectrode determination (Figure 4). The zero depth in the oxygen profiles represented the calculated sediment-water interface. Oxygen concentrations all exhibited a distinct and steep decrease with depth just below the sediment surface, gradually reaching zero at a sediment depth of 1.5–8.0 mm, indicating oxygen consumption in the sediments. Penetration depths of oxygen ranged from 1.26 mm to 6.23 mm with an average of 3.06 mm. The porosities of sediments were within the range 0.43–0.90.

**Figure 5:**
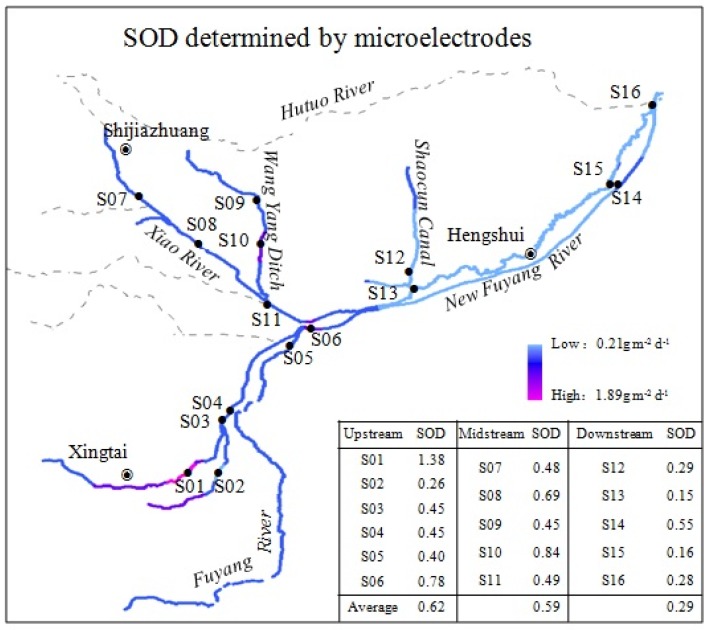
Spatial variations of the sediment oxygen demand determined by microelectrodes in the Ziya River Watershed.

Rates of SOD measured by microelectrode methods and its distribution are presented in Figure 5. SOD rates measured by microelectrodes corrected to 20 °C ranged from 0.15 to 1.38 g/(m^2^·d), with a mean value of 0.51 g/(m^2^·d). Maximum SOD was found at S01 and minimum SOD was found at S13, which was the same as the results derived from core incubations. The maximum SOD was a factor of nine larger than the minimum SOD. The average flux in the upstream part of the watershed was 0.62 g/(m^2^·d), higher than that in the midstream (0.59 g/(m^2^·d)) and that in the downstream (0.29 g/(m^2^·d)).

### 3.3. Comparison between Incubations and Microelectrodes

**Figure 6:**
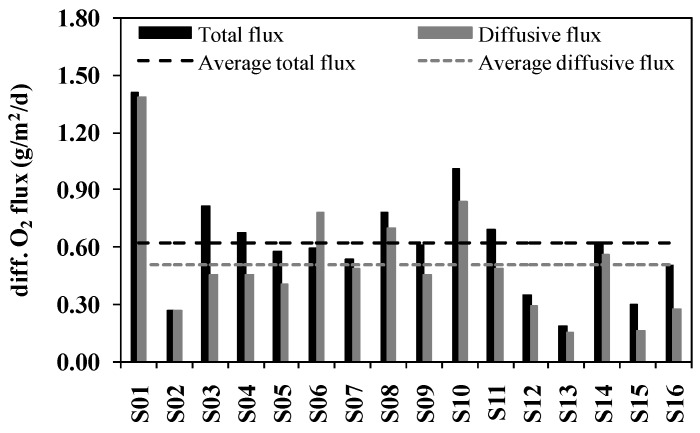
Sediment oxygen demand measured during August 2003. Total flux referring to SOD determined by core incubation and diffusive flux referring to SOD determined from microelectrode estimation.

**Figure 7:**
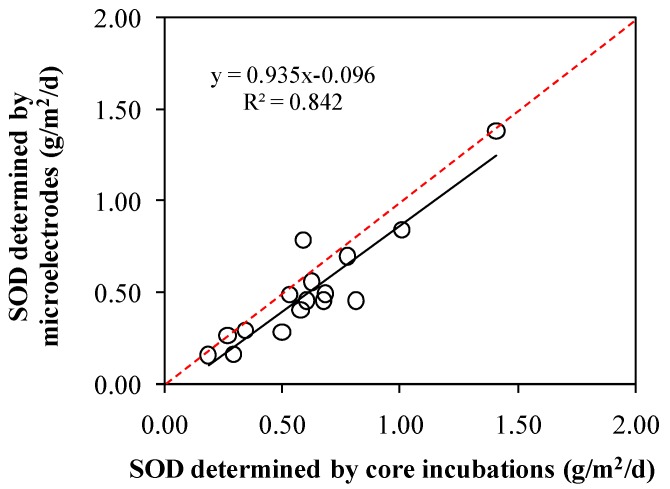
Comparison of SOD determined by core incubations and by microelectrodes. The red dotted line represents the ideal value when the two methods are identical.

The effect of measurement technique on laboratory core incubationor microelectrode based SOD values was not known. This paper compared the two measurements designed to evaluate and provide a basis for judging the utility of laboratory core measurement microelectrodes as surrogates for each other. Core incubations provided a measurement of the total flux. Oxygen microprofiles were applied to the calculation of the diffusive flux. A small difference was observed between total fluxes and diffusive fluxes (Figure 6). The two techniques used during this study to estimate the SOD (core incubations and microelectrode profiles) both provide approximate results. In this study, total fluxes were a little higher than diffusive fluxes at most of the sampling sites (Figure 6). The average total flux was 0.62 g/(m^2^·d), while the average diffusive flux was 0.51 g/(m^2^·d). The total flux was a factor of 1.2 larger than the diffusive flux. SOD determined by core incubations was shown to strongly correlate with that determined by microelectrodes (Figure 7, *R*^2^ = 0.842). SOD determined by microelectrodes can explain 93.5% of SOD determined by core incubations (Figure 7, k = 0.935). Previous studies have found a k value of 0.407 with a *R*^2^ of 0.785 [28], and a k value of 0.900 with a *R*^2^ of 0.755 [29]. The correlation of the results of the two methods used in this study is higher than that shown in the aforementioned studies.

Statistical analysis methods of Wilcoxon signed-rank test was applied to infer the conformity or the deviation between the two techniques. The statistical analysis result clearly showed that the microelectrode method is producing smaller results than the total flux method—in 15 of 16 sites—and the diffusive flux was smaller than the total flux. *Wilcoxon* signed-rank test showed that the data accepted by the two methods had significant difference (*p* < 0.05). Although some of the data given in Figure 7 showed relatively good agreement among the two techniques, methodological differences were identified as the biggest source of variations in the data.

Possible reasons for the observed a little difference between the diffusive and total flux estimates were considered. The processes taken into account by the two techniques are different. The total flux estimated by the core incubation typically integrates all processes linked to oxygen transfer: biological respiration of all living organisms, diffusion and advective movement of pore water induced by faunal activity (e.g., activepumping in burrows, biodiffusion because of the erratic movements of the fauna), and chemical oxidation of reduced substances in the sediment. The diffusive flux estimated from the microelectrode profile assumes that the transport of chemical species is accomplished by molecular diffusion excluding the other transport processes [28]. Based on this interpretation, the total flux is generally higher than diffusive flux and the inclusion of these processes in our calculation should explain the difference between the total and diffusive oxygen demand.

The findings of this study suggest that there is some calibration relation between the laboratory incubation method and microelectrode method for SOD measurement in this research region. SOD can be measured through laboratory or *in situ* methods; however, both approaches have limitations. For the choice of laboratory core methods and microelectrodes, laboratory variables can be controlled more easily in the laboratory core incubations, such as temperature, light, degree of agitation, and DO concentration. In the research mechanism aspects, the microelectrode method would have more advantages. Confirming the accuracy of determination results through validating one measurement with another measurement is not a common practice but considered to be meaningful. A little difference between the results determined by the two methods indicated that both methods could be accepted as general methods.

### 3.4. Effects of Point and Non-Point Pollutions on SOD

SOD is the result of bacterial activity and chemical processes by which organic material is decomposed. Those nutrients and organic matters loading from exogenous sources may accelerate DO depletion and hypoxia [30]. The sources of these organic materials include deposits from streams, constituents added from point and non-point sources, and biotic deposits from stream biota. Actual contributions of point and non-point source pollutions to river can be determined based on total flux partition across river sections. Using the investigated data on pollution sources from 2011 to 2015 (organized and carried out by our team), we analyzed the relative contributions of point source pollution and non-point source pollution to COD (Chemical Oxygen Demand) and ammonia in the overlying water. The result indicated that the pollution of surface water in the Ziya River Watershed is mainly the point source pollution, and its contributions to COD and ammonia both accounted for about 60%–80%. Industrial pollution sources contributed nearly 80% to COD and 50% to ammonia. About 142 thousand tons of COD and 10.1 thousand tons of ammonia per year were discharged into the Ziya River Watershed.

Table 1 showed the contributions of point and non-point source pollutions to COD and ammonia. In the upstream, 65% of COD and 21% of ammonia were discharged from industrial pollution sources, and mainly produced by papermaking industry and paper packing industry. In the midstream, 42% of COD and 33% of ammonia were discharged into the Xiao River from industrial pollution sources, and mainly produced by the pharmaceutical industry and petrochemical industry. In Wang Yang Ditch, COD and ammonia were almost entirely produced by pharmaceutical industry. In the downstream, COD and ammonia were almost entirely produced by leather industry and were discharged into Shaocun Canal. About 30% of COD and 20% of ammonia were discharged into Fuyang River from industrial pollution sources, and mainly produced by petrochemical industry. 38% of COD was produced by non-point source pollution as livestock and poultry breeding. Those exogenous contaminants accelerated organic materials depositionin sediment accompanied with suspended particles.

The point and non-point pollution introduced lots of COD, ammonia, nutrients, and heavy metals to the Ziya River system. Based on the data accepted in our early study [31,32], a Spearman regression analysis was conducted for analyzing the relative contributions of water quality indicators to SOD in the Ziya River Watershed (Table 2). Significant correlation between SOD with COD was found (*p* < 0.05). The main reason for this result may be a mass of COD deposition and accumulation in sediment caused the high sediment oxygen consumption. Correlations between SOD with TP (Total Phosphorus) and SRP (Soluble Reactive Phosphorus) (*p* < 0.05) were also significant. That may be because overload P promoted eutrophication and algal bloom, and algal residuals were the main souse of organic matters. Those exogenous contaminants accelerated organic matters deposition in sediment accompanied with suspended particles. Thus, a lot of contaminant accumulation in sediment caused by deposition may be the main reason of high SOD in the Ziya River Watershed.

### 3.5. Effects of SOD on the River System

The ranges of SOD rates observed by a number of investigators, including those determined by core incubations in this study are presented in Table 3. The results in our study compared favorably with those observed for other water bodies with similar heavy pollution characteristics. These data show that contaminated waters have a comparatively high SOD value. The SOD value is usually less than 0.5 g/(m^2^·d) when the water body is clean and uncontaminated. Early research reported a few mg/(m^2^·h) for sand and larger than 100 mg/(m^2^·h) for organically-enriched sediment. The USEPA uses values below 1.0 g/(m^2^·d) as low values andabove 1.0 g/(m^2^·d) as high values.Previous researchhas indicated that there is a decreased oxygen consumption rate asoxygen concentration in the overlying water becomes limiting (below about 2 mg/L) [8]. If the DO concentration in the overlying water goes to zero, the SOD will cease. The mean DO concentration in the Ziya rivers is always below 3 mg/L, even less than 2 mg/L. Sediments had been in anaerobic conditions for a long time. The SOD rates in the real environment were at low levels or zero. That is also the reason of the *in situ* method not used in this study, so the SOD rates determined by the two laboratory methods could not reflect the real condition of sediment oxygen consumption in the Ziya river watershed. It is possiblythey could reflect the high potentialoxygen uptake of sediments. However, when water pollution control projects took effect, SOD rates would increase as the water quality improved. The potential risk of further severe sediment oxygen depletion, even after the water quality improvement, should be taken seriously in water pollution control performances.

Appropriate concentration of dissolved oxygen is necessary for the survival of aquatic organisms. Previous work in this areahas always focused on the oxygen consumption by organics or ammonia, yet has not adequately dealt with the sediment oxygen demand. In the case of 5 mg/LDO restored in the overlying water with average water deep of 0.63 m in the Ziya rivers, the DO will be consumed in 4.8 days using the average SOD rate of 0.62g/(m^2^·d). With the maximumSOD rate of 1.41g/(m^2^·d), the DO will be consumed in 2.4 days.The influence of these SOD rates on water column DO concentrations is prominent. When the pollution sources have been cut off and water quality has been improved, yet contaminated sediments had not been restored, SOD would greatly affect DO level in rivers. This potential risk merits further detailed attention.

The two methods discussed in this study measured the potential oxygen uptake of sediments in the laboratory. SOD is not exerted until oxygen in the overlying water column penetrates into contact with the oxygen-consuming constituents of the sediment. Contactoccurs by oxygen diffusion down to the sediment and/or by the movement ofoxygen-consuming species in the sediment up into the oxygenated water column. Many nutrients and heavy metals have a high affinity for sediment [38]. Movement of these materials from the sediment to the overlying water is related to SOD processes occurring at the sediment–water interface [39,40]. When DO in the overlying water is recovered, the sediment budget will initiate a strong oxygen depletion effect and nutrient and heavy metal problems.

## 4. Conclusions

To investigate the sediment oxygen demand, a detailed study was conducted using two different methods at 16 stations in the Ziya River Watershed, China. The use of the two different methods, laboratory core incubations and microelectrodes, allowed us to determine the degree and extent of the SOD in the Ziya River watershed and compare total and diffusive oxygen fluxes. The primary conclusion of this study is SOD rates for the Ziya River Watershedaveraged 0.62 g/(m^2^·d) and had a 0.19–1.41 g/(m^2^·d) range when measured using core incubations, and had an average of 0.51 g/(m^2^·d) and range of 0.15–1.38 g/(m^2^·d) when measured usingmicroelectrodes. The SOD rates determined bycore incubationswere well correlated with those determined by microelectrodes. *Wilcoxon* signed-rank test suggested that the two methods had significant difference (*p* < 0.05). The microelectrode method producd smaller results than the incubation method. The SOD found in this study was in the same order of magnitude as previously-reportedoxygen fluxes. Although SOD rates determined in this study could not reflect the real condition, they showed potentially high oxygen depletion when DO increased. This study would be helpful for preventing the potional risk of oxygen depletion and nutrient release during river restoration.

## Figures and Tables

**Table 1:** Analysis of the point and non-point pollution in the Ziya River Watershed.

River		Sample Site	Pollutants Quantity Inlets Into Rivers		Contributions of Industrial Pollution Sources	
			COD (10^3^ t)	Ammonia (t)	COD	Ammonia
Upstream	Shunshui River	S01	33.00	0.16	65%	21%
	South Li River	S02				
	Li River	S03–S05				
Midstream	Xiao River	S07, S08	39.00	0.48	42%	33%
	Wang Yang Ditch	S09, S10	2.30	0.15	~100%	~100%
Downstream	Shaocun Canal	S12, S13	4.50	0.52	~100%	~100%
	Fuyang River	S14–S16	34.00	2.03	30%	20%

**Table 2:** Results of Spearman regression analysis.

	TN	NH_3_-N	NO_3_^−^-N	NO_2_^−^-N	TP	SRP	SOD	COD
TN	1.000	0.915 **	0.291	0.249	0.382	0.225	0.350	0.333
NH_3_-N		1.000	0.079	0.124	0.285	0.219	0.006	0.056
NO_3_^−^-N			1.000	0.411	0.253	−0.118	0.441	0.212
NO_2_^−^-N				1.000	0.003	−0.310	0.182	0.246
TP					1.000	0.770 **	0.837 **	0.077
SRP						1.000	0.721 **	0.174
SOD							1.000	0.642 **
COD								1.000

** significant in 99% confidence intervals.

**Table 3:** ComparativeSOD values reportedby various researchers.

Pollution Condition	Range of SOD at 20°C (g/(m^2^·d))	Location	References
Admittingabundantdomestic wastewater, severe eutrophication	0.48–1.44	man-madeLake Ton-Ton, Uruguay	[33]
summertime occurrences of anoxia/hypoxia	0–1.64	Chesapeake Bay, USA	[34]
Low DO, ~4 mg/L	0.47–1.28	Tolo Harbor, Hong Kong	[9]
municipal andagricultural wastewater, BOD: 2–5 mg/L	0.13–1.36	Arroyo Colorado River	[8]
urban sewage and industrial wastewater pollution, hypoxia	0.24–1.58	Keelung River, Taiwan	[35]
Low DO (less than 1 mg/L), very high nutrient concentrations in the river	0.37–1.25	Xindian River, Taiwan	[36]
admitting paper industry wastewater	0.22–1.82	Athabasca River	[37]
Low DO, ~3 mg/L, admitting abundant domestic and industrial wastewater, severe hypoxia	0.19–1.41	the Ziya River Watershed, China	This study
